# Fly Genes That Help Devour a Fungal Parasite

**DOI:** 10.1371/journal.pbio.0040018

**Published:** 2005-12-20

Faced with a world full of potentially harmful bacteria, fungi, and viruses, multicellular organisms have evolved efficient innate mechanisms to combat infection soon after a pathogenic exposure. Many aspects of this innate response are shared by organisms as diverse as flies and humans, pointing to a common and ancient origin for these innate defenses. While humans and other vertebrates possess adaptive immunity—which can recognize billions of different pathogens and mount better responses with each exposure to a pathogen—flies and other invertebrates must rely only on innate immunity.

As early as 1882, Élie Metchnikoff discovered that cells in starfish and water fleas ingested and destroyed microbes as part of the immune response. (The Russian zoologist and microbiologist would receive the Nobel Prize for his discovery.) Only recently have scientists realized how many different organisms use this strategy, called phagocytosis, to protect themselves. When a pathogen is detected, signaling pathways within the phagocytic cell reconfigure the cytoskeleton and send out pseudopodia protrusions that engulf the pathogen. While these morphological and structural changes have been well described, few studies have outlined the cellular components driving this process. In a new study by Shannon Stroschein-Stevenson et al., the labs of Patrick O'Farrell and Alexander Johnson turned to the fruit fly, Drosophila melanogaster—a well-established model organism for both genetics and innate immunity—to explore the phagocytosis of the fungus Candida albicans, a widespread human pathogen. [Fig pbio-0040018-g001]

**Figure pbio-0040018-g001:**
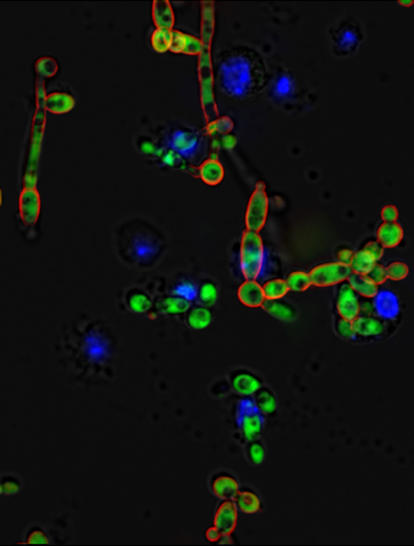
Drosophila cells engulf the human pathogen Candia albicans, tagged with green fluorescent protein

Using the S2 cell line, which is derived from flies and shares many properties with the plasmatocytes that perform phagocytosis in the fly, the authors used RNA interference (RNAi) to conduct a global search for genes related to phagocytosis. They identified several genes specifically dedicated to dispatching C. albicans, then focused on one gene whose function in the fly was unknown. The gene, called *Macroglobulin complement related (Mcr)*, is closely related to human proteins that activate what's known as the complement cascade, an ancient mechanism that flags pathogens for subsequent recognition by phagocytic cells. The authors show that Mcr is closely related to four fly proteins (members of the thioester protein [Tep] family), and that these proteins act on different pathogens, functioning as part of a “primitive complement system” that targets specific pathogens.

Stroschein-Stevenson et al. first mixed fluorescently tagged C. albicans with the S2 cells and observed the S2 cells ingest the fungal cells, confirming that the cell line can phagocytose the fungus. Next, they screened the cells for phagocytosis defects using RNAi; RNAi disrupts the function of a target gene by using short lengths of double-stranded RNA (dsRNA) with complementary sequences to that gene to force the degradation of the gene's messenger RNA and prevent its translation into protein. The authors used a library of over 7,000 dsRNAs corresponding to most of the fly's conserved genes to screen for phagocytosis gene candidates. After treating the S2 cells with dsRNAs, they mixed the cells with the fluorescently tagged fungus to visualize the effects on phagocytosis.

The initial screen flagged some 400 dsRNAs that decreased fungal phagocytosis. A few more screens excluded dsRNAs that were likely either false positives or indirectly involved, leaving 184 dsRNAs that impaired phagocytosis. The screen identified many known phagocytosis-related genes that had been picked up in earlier screens (for example, the cytoskeletal protein actin and its various regulators, which help form the active membrane protrusions required for phagocytosis), but it also turned up many other genes not previously implicated in the process.

To distinguish genes specific to C. albicans from those with a broader role in phagocytosis, the authors repeated the screen with two different “challengers,” Escherichia coli and latex beads. Most of the 184 dsRNAs impaired phagocytosis of all three targets, but only a few specifically disrupted C. albicans phagocytosis, including the dsRNA for Mcr. Additional experiments confirmed Mcr's specificity for C. albicans. Since Mcr is closely related to a family of four fly Tep proteins, the authors tested whether each protein is required for the phagocytosis of three distinct pathogens: C. albicans, E. coli, and Staphylococcus aureus. Again, impaired phagocytosis of C. albicans occurred only when Mcr was disabled. (Adding Mcr proteins to the S2 growing medium restored the cells' ability to phagocytose C. albicans.) E. coli phagocytosis was reduced only with TepII disruption, and S. aureus phagocytosis slowed only with TepIII silencing.

The authors go on to show that S2 cells secrete Mcr, which then binds to C. albicans (but not to a closely related fungus) and promotes phagocytosis. Altogether, these results show that the fly's innate immune system dispatches specialized proteins to destroy specific pathogens. The 184 genes identified in this screen should prove a valuable resource for investigations into the cellular components of an ancient defensive strategy. And with the visual screen described here, even researchers without the latest automated equipment can get started right away. *—Liza Gross*

